# Antioxidant Activity, Total Phenolic Content, Individual Phenolics and Physicochemical Parameters Suitability for Romanian Honey Authentication

**DOI:** 10.3390/foods9030306

**Published:** 2020-03-08

**Authors:** Daniela Pauliuc, Florina Dranca, Mircea Oroian

**Affiliations:** Faculty of Food Engineering, Stefan cel Mare University of Suceava, 720225 Suceava, Romania; daniela_pauliuc@yahoo.com (D.P.); florina.dranca@usm.ro (F.D.)

**Keywords:** honey, authentication, physicochemical parameters, PCA

## Abstract

The present study aimed to evaluate the physicochemical characteristics of honey (raspberry, mint, rape, sunflower, thyme and polyfloral) produced in Romania. The honey samples were from the 2017 to 2018 harvest and were subjected to melissopalynological analysis, alongside the determination of the following physicochemical parameters: moisture content, pH, free acidity, electrical conductivity (EC), hydroxymethylfurfural (HMF) content, color, total polyphenols content (TPC), flavonoids content (FC), DPPH radical scavenging activity, phenolic acids, flavonols, sugars and organic acids in order to evaluate the usefulness of this parameters for the classification of honey according to botanical origin. The results of the melissopalynological analysis revealed that five types of honey samples had a percentage of pollen grains above the minimum of 45%, which was required in order to classify the samples as monofloral honey. The total polyphenols content reached the maximum value in the case of dark honey such as mint honey, followed by raspberry, thyme and polifloral honey. Fructose, glucose, maltose, sucrose, turanose, trehalose, melesitose, and raffinose were identified and quantified in all samples. Gluconic acid was the main organic acid in the composition of all honey samples. Principal component analysis (PCA) confirmed the possibility of the botanical authentication of honey based on these physicochemical parameters.

## 1. Introduction

Honey is used both as medicine and a food source [1] and it is defined, according to Codex Alimentarius and EU Directive 110/2001 [2,3], as a sweet natural substance produced by bees (*Apis melifera*) from nectar or from the secretions of some plants, which is collected by bees and transformed by combining specific substances [4]. Honey is a complex food product, which is derived from nature and is the only natural sweetener that humans can use without processing [5], and therefore is very important economically [6].

Honey has a very complex chemical composition because it contains about 80% sugars, of which an important part is represented by glucose and fructose, 15–17% water, 0.1–0.4% protein and other compounds that are quantified as ash 0.2% [7,8]. In addition, honey also contains, in small quantities, about 200 other constituents, which include amino acids, phenolic compounds, organic acids, vitamins, minerals, and enzymes [9]. This multitude of minor components can be added by bees or comes directly from nectar due to the ripening process [10,11].

The chemical composition depends on the source of honey, which refers to the botanical and geographical origin, as well as the environmental conditions [12]. Monofloral honey is increasingly required on the market and it is necessary to be able to determine some parameters regarding the authentication of the botanical and geographical origin. Monofloral honey is more expensive than polyfloral honey; honey labeled as having a certain floral origin must come entirely or largely from the specific floral source and exhibit the organoleptic, physicochemical and microscopic characteristics of the honey source, as provided in international food standards [2,11].

Considering that bees feed on various plants, pure monofloral honey is generally very rare. The identification of the origin of honey and the proof of its authenticity has become an important problem with the globalization of the honey market, involving about 150 countries [13]. The interest in identifying the floral origin of honey has increased in recent years due to the high preference of consumers for certain types of honey. Consumer preferences often vary depending on different sensory perceptions and medicinal properties. Thus, numerous research has been published to date, which aimed to develop reliable methods for indicating the floral origin of honey [14].

Pollen analysis can be successfully used for the identification of the floral origin of honey. Therefore, melissopalinology should usually be supplemented by physicochemical and organoleptic analysis. Thus, to classify honey by botanical origin, a global interpretation of all results is required [15]. The melissopalynological analysis consists of counting the pollen grains and classify the honey according to its principal pollen grain percentage, for some honey such as sunflower, raspberry, rape and mint the principal pollen must reach at least 45% of the total pollen grains [16] while for thyme honey the *Thymus* spp. pollen grains must be at least 18% of the total pollen grains [17].

Therefore, new analytical methodologies were used to determine the botanical origin; these include the chromatographic, spectroscopic, e-tongue and molecular biological methods [18,19]. Physicochemical parameters (color, moisture, acidity) can vary widely in different types of honey and this contributes, to a certain extent, to their organoleptic characteristics. This is the reason why chromatographic techniques are more eloquent in the classification of honey and special attention should be paid to identifying certain specific minor components [20]. In addition to the classical techniques used to authenticate honey, the use of DNA-based methods for pollen identification has also spread. DNA-based identification has the potential to reduce processing time and increase the level of discriminated species [21]. Soares et al. [22] reported that they extracted the DNA markers and the yield and purity of the extracts were evaluated by UV spectrophotometry; this method was validated successfully with honey of known origins and applied to the entomological authentication of 20 commercial samples from different European countries.

Spectroscopic techniques, such as Fourier transform infrared spectroscopy (FTIR) and Raman spectroscopy, are alternative methods for authenticating honey and these techniques are reliable, practical and not time-consuming. FTIR spectroscopy is sensitive to the chemical composition of the sample, and when coupled with multivariate statistical analysis, it provides accurate results in determining the botanical origin of honey [9]. Svecnjak et al. [23] used FTIR-ATR spectroscopy to confirm the botanical origin of collected honey samples from beekeepers from different Croatian regions. Rheology and electrical tongue are also part of the alternative methods of authentication of honey. The voltammetry technique implies a high sensitivity and the electronic tongue can be regarded as a reference system in honey authentication [24]. Sousa et al. [25] reached a 100% correct classification of chestnut (*Castanea* spp.), lavender (*Lavandula* spp.) and raspberry honey (Rubus spp.) with a potentiometric electronic tongue. The exact classification was obtained after honey samples were separated according to their color and then the authentication of each type of honey was done on their botanical origin.

NMR is a fingerprint technique that is used to obtain information about the structure of components [26]. Spiteri et al. [27] analyzed 816 honey samples from 60 different botanical origins by the NMR technique and observed specific profiles for the botanical sources of origin.

In this study, melissopalynological analysis and analysis of physicochemical parameters (moisture content, pH, free acidity, electrical conductivity, hydroxymethylfurfural content, color, total polyphenols content, flavonoids content, DPPH radical scavenging activity, phenolic acids, flavonols, sugar composition, organic acids compositions) was performed to authenticate the botanical origin of sunflower, raspberry, thyme, mint, rape and polyfloral honey from Romania.

## 2. Materials and Methods

### 2.1. Honey Samples

Forty-five honey samples from the flowering season of 2017 to 2018 were purchased from beekeepers or apicultural associations from different regions in Romania.

Honey samples were kept away from sunlight at room temperature until the analysis. Thyme (*Thymus* spp.), rape (*Brassica* spp.), mint (*Mentha piperita*), raspberry (*Rubus idaeus*), and sunflower (*Helianthus* spp.) honey were of interest for this research.

### 2.2. Melissopalynological Analysis

Melissopalynological analysis was carried out according to Louveaux and Vorwohl [28]. The pollen was examined under a microscope using ×40 magnification on a Motic microscope (Motic, Xiamen, China). For achieving the botanical origin at least 800 pollen grains were counted.

### 2.3. Physicochemical Analysis

#### 2.3.1. Moisture Content, pH, Free Acidity, HMF Content and Electrical Conductivity

Moisture content, free acidity, pH, HMF content and electrical conductivity were determined according to the methods of the International Honey Commission [8,29].

#### 2.3.2. Color

Honey color analysis was performed using the Pfund scale (Pfund HI, Hanna Instruments, USA) and CIEL*a*b* coordinates (portable chromameter CR-400, Konica Minolta, Tokyo, Japan), respectively.

#### 2.3.3. Determination of Total Phenolic Content

The method proposed by Biesaga et al. [30] was used to determine the total phenolic content (TPC) and sample preparation was made, as follows: 1 g of honey sample was extracted with 5 mL of 40% methanol/acidified water (*v/v*, pH = 2, HCl). Then, the samples were stirred for 15 min with a magnetic stirrer. From the extract, 0.2 mL was mixed with 2 mL of Folin–Ciocalteu reagent 1:10 and 1.8 mL Na_2_CO_3_ 7.5% (*w/v*). The samples were kept in the dark for 20 min and the absorbance was measured at 750 nm using a UV-NIR spectrometer HR4000CG-UV-NIR (Ocean Optics, St. Petersburg, FL, USA). Gallic acid solutions with concentrations ranging from 0–400 mg·L^−1^ were used to obtain the calibration curve.

#### 2.3.4. Determination of Flavonoids

From the extract prepared as presented in Section 2.3.3, 5 mL were mixed with 300 µL of NaNO_2_ 5% (*w/v*) and 300 µL of AlCl_3_ 10% (*w/v*) [30]. After 5 min in the dark, the samples were mixed with 2 mL of NaOH 1 N. The samples were kept for 6 more minutes in the dark and then the absorbance of each sample was read at 510 nm with a HR4000CG-UV-NIR spectrometer. Quercetin solutions with concentrations ranging from 0–10 mg·L^−1^ were used to obtain the calibration curve.

#### 2.3.5. DPPH Assay

The determination of 1,1-diphenyl-2-picrylhydrazyl (DPPH) radical scavenging activity required the following sample preparation: 1 g of honey was dissolved in 5 mL of methanol 40% (*v/v*, with acidified water) and stirred for 15 min with a magnetic stirrer [31]. Then, 35 µL of honey solution was mixed with 250 µL of DPPH. The absorbance was measured at 515 nm using a QE65000 spectrometer (Ocean Optics, St. Petersburg, FL, USA). The results were expressed as % DPPH using the formula in Equation (1):

(1) $$\%\;\mathrm{DPPH}\;=\;\left(A_{0}-\frac{A_{1}}{A_{0}}\right)\times 100,$$

where *A*_0_ is the DPPH absorbance, *A*_1_ is the sample absorbance.

#### 2.3.6. Determination of Sugars Composition

Sugars composition was determined according to the IHC (International Honey Commission) methods [8,29]. The samples were filtered through 0.45 µm PTFE membrane filters prior to the injection in the HPLC instrument (Schimadzu, Kyoto, Japan) equipped with a LC-20 AD liquid chromatograph, SIL-20A auto sampler, CTO-20AC column oven, and RID-10A refractive index detector. The separation was performed on a Phenomenex Luna^®^ Omega 3 μm SUGAR 100 Å HPLC Column 150 × 4.6 mm. Peaks were identified based on their retention times and the determination of sugar content was made according to the external standard method on peak areas or peak heights. The mobile phase was acetonitrile:water (80:20, *v/v*), with a flow rate of 1.3 mL·min^−1^; column and detector temperature was 30 °C and the sample volume injection was 10 µL. Standard solutions of fructose, glucose, maltose, sucrose, turanose, trehalose, melesitose, and raffinose were individually injected to calculate the sugar content of each honey sample by using peak areas based on the retention time.

#### 2.3.7. Determination of Polyphenols Composition

Honey solutions were prepared following the steps presented in Section 2.3.3 [30]. The samples were filtered through 0.45 µm PTFE membrane filters and then injected (with a volume of 10 µL) into the HPLC instrument (Schimadzu, Kyoto, Japan) for analysis using an SPD-M-20A diode array detector. The separation was carried out on a Phenomenex Kinetex 2.6 μm Biphenyl 100 Å HPLC Column 150 × 4.6 mm thermostated at 25 °C. Elution was carried out with a solvent system consisting of 0.1% acetic acid in water (solvent A) and acetonitrile (solvent B) as previously described by Palacios et al. [32] with modifications. The solvent flow rate was of 1 mL·min^−1^. The determined phenolic compounds were gallic acid, vanillic acid, protocatechuic acid and *p*-hydroxibenzoic acid at 280 nm, and chlorogenic acid, *p*-coumaric acid, caffeic acid, rosmarinic acid, myricetin, quercetin, luteolin and kaempherol at 320 nm. The obtained standard calibration curves showed high degrees of linearity (*R*^2^ > 0.99). Data collection and subsequent processing were performed using the LC solution software version 1.21 (Shimadzu, Kyoto, Japan).

#### 2.3.8. Determination of Organic Acids Composition

The method used to determine the organic acids involved a sample preparation of 0.5 g of honey mixed with 2.5 mL of 4% metaphosphoric acid (*w/v*), then the samples were vortexed. After, the samples were centrifuged for 5 min at 3500 rpm using a Z216-MK refrigerated centrifuge (Hermle Labortechnik, Wemingen, Germany) [33]. The sample was injected in the HPLC instrument (Schimadzu, Kyoto, Japan) with a diode array detector. The separation was carried out on a Phenomenex Kinetex^®^ 5 μm C18 100 Å HPLC Column 250 × 4.6 mm. The mobile phase used was a mixture of 0.5% metaphosphoric acid and acetonitrile (50/50, *v/v*) at a flow rate of 0.8 mL·min^−1^. The volume of injection was 10 µL. The organic acids identification and quantification were carried out at 210 nm. The organic acids that were determined were acetic acid, lactic acid, propionic acid, butyric acid, and gluconic acid. The concentration of organic acids was expressed as mg/L.

### 2.4. Statistical Analysis

The analysis of variance (ANOVA) (LSD (least significant difference) test and α = 0.05 were applied) and principal component analysis (PCA) were used for achieving the suitability of the analyzed parameters for the botanical authentication of honey. ANOVA was carried out using Statgraphics Centurion XVIII software—trial version (Manugistics Corp., Rockville, MD, USA), while PCA was carried out using Unscrambler X version 10.1 (Camo, Norway), respectively.

## 3. Results

### 3.1. Melissopalynological Analysis

Melissopalynological analysis is considered a traditional approach for the determination of the botanical origin of honey, and is a method of analysis that involves microscopic examination of pollen grains in order to identify the plants that were visited by bees during honey production [34]. Pollen analysis is a method developed and proposed by the International Bee Botanical Commission (IBBC) in 1970, which was revised in 1978 [28]. A honey sample can be classified as monofloral honey when more than 45% of the pollen grains belong to a single plant species for rape, raspberry, mint and sunflower [16], while thyme honey must be at least 18% total pollen grains [17]; this type of honey is the most preferred by consumers for its specific aroma, taste and biological properties [18,19]. The melissopalynological analysis is presented in Table 1. The raspberry honey had the principal pollen *Rubus idaeus* (49.1–82.3%), rape honey had the principal pollen *Brassica spp.* (50.1–71.1%), sunflower has the principal pollen *Helianthus* spp. (46.5–92.1%) and thyme had the principal pollen *Thymus* spp. (22–45%), respectively.

Based on pollen analysis, the samples were classified according to the botanical origin as raspberry (6 samples), rape (10 samples), sunflower (9 samples), thyme (4 samples), mint (10 samples) and polyfloral honey (6 samples). Of the monofloral honey samples, the highest percentage of pollen grains was found in rape and sunflower honey. 

### 3.2. Moisture Content

The moisture content of honey is dependent on factors such as the relative humidity in the region where honey comes from and the processing and storage conditions [11]. The Codex Alimentarius standard established that the moisture content of honey must be below 20% [2]. Honey samples that do not meet this criterion could become unstable during storage and thus be susceptible to deterioration by fermentation caused by yeast and bacteria naturally found in honey [35]. The moisture content of the analyzed samples ranged between an average value of 17.36% in thyme samples and a maximum average value of 19.60% in the polyfloral honey samples, as shown in Table 1. The botanical origin of honey did not influence the variation of the moisture content (*p* > 0.05), while the year of honey production had some influence (*p* < 0.05) on this parameter. All honey samples had moisture contents in the limits established by legislation. The mint and thyme honey samples had values between 17.36% (thyme honey) and 17.77% (mint honey), while Boussaid et al. [36] reported for mint honey the value of 19.8% and for thyme honey, 18.16%. The results of this analysis were in accordance with the values reported by Karabagias et al. [17] who determined in honey samples a moisture content that ranged from 10.74% (Symi honey sample) to 20.94% (Lakonia honey sample). Mărghitaș et al. [37] reported a variation in the moisture content for the Romanian honey between 16.6% and 20%, while Küçük et al. [38] reported values of moisture content from 19% to 19.7% for Anatolian honey. Escuredo et al. [39] determined a moisture content between 15.5% and 19.8% for honey samples from Spain.

### 3.3. pH

Honey has a pH that usually varies between 3.5 and 5.5 and is dependent on the compositions of organic acids, which are chemical components that give the aroma of honey and at the same time protect it against microbiological damage. Therefore, the pH can be considered an indicator of potential microbial growth, as a value of 7.2 to 7.4 is optimal for the development of most microorganisms. The average pH values of the honey samples ranged from 3.91 in the case of thyme honey to a maximum of 4.22 in the case of rape honey (Table 2). 

Differences in this parameter were determined by the botanical origin of honey (*p* < 0.05), but not by year (*p* > 0.05). Romanian mint and thyme honey samples had pH values similar to Tunisian mint and thyme honey [36]. In Tunisian mint honey samples, the pH value was 4.11 [36], while in Romanian mint honey, it was 4.20. For thyme honey, the pH value was 3.87 (Tunisian honey) and 3.91 (Romanian honey).

Therefore, a pH between 3.2 and 4.5 was considered acceptable for honey samples [11] and the pH values determined for the studied samples were within this range. The pH of honey is of particular importance during the extraction and storage of honey because of its influence on the texture, stability and storage time [5]. The average pH values of honey samples from Vojvodina (Serbia) ranged between 3.88 (sunflower honey) and 3.99 (acacia honey) [35].

### 3.4. Free Acidity

The free acidity of honey is determined by the presence of organic acids and other compounds such as esters, lactones and inorganic ions found in its composition [11]. Contribution to this parameter also presents the composition of protein, phenolic acids and vitamin C, which are chemical components that act as H^+^ donors [40]. Determining the acidity helps to appreciate the freshness of the honey. As the composition of honey deteriorates, an increase of free acidity occurs as a result of the fermentation of sugars into organic acids. According to the EU legislation [41], for this parameter a maximum of 50 milliequivalents of acid per 1000 g is allowed [2]. In our study, the highest acidity was determined in sunflower (31.63 meq·kg^−1^) honey and the lowest (16.01 meq·kg^−1^) in rape honey (Table 2). The botanical origin of honey had a significant influence on this parameter (*p* < 0.01), while the year of production determined no significant variation between samples (*p* > 0.05). Lazarević et al. [42] observed similar results, they determined the highest free acidity (27.2 meq/kg) in sunflower honey and the lowest values of the parameter (11.6 meq·kg^−1^) in acacia honey. Significant differences in the function of the botanical origin of honey were also reported for the free acidity of acacia and hay honey [43]. Oroian and Ropciuc [44] reported that free acidity varied between 6.63 meq·kg^−1^ in tilia honey, 13.02 meq·kg^−1^ in sunflower honey and reached the maximum value in the case of polifloral honey (20.83 meq·kg^−1^).

### 3.5. HMF Content

The HMF content is a chemical parameter that can be used to study the degree of freshness of honey and consequently its degree of deterioration. The causes of honey deterioration could be due to strong or prolonged thermal treatment and inadequate storage conditions [45]. As seen in Table 2, honey samples had an HMF content between a minimum of 8.26 mg HMF·kg^−1^ (sunflower honey) and a maximum of 50.8 mg HMF·kg^−1^ (thyme honey). Botanical origin had a significant influence (*p* < 0.001) on this parameter. For some of the samples of mint (two samples) and thyme honey (one sample) that were analyzed, the HMF content was above the maximum concentration (40 mg·kg^−1^) allowed by European legislation [41]. In the case of these samples, it is possible that there was an overheating during processing and/or storage, which might have influenced the HMF content in honey.

Rodríguez et al. [46] observed that the avocado honey had a maximum level of HMF of 27.1 mg·kg^−1^. Another study, focused on the quality of honey from Rio Grande do Sul State (Brazil), reported values of 0.47–22.72 mg HMF·kg^−1^ of honey, which met the quality requirements established by both Brazilian legislation (upper limit of 60 mg HMF·kg^−1^) and international standards (Codex, 2001—maximum of 40 mg HMF·kg^−1^) [47].

### 3.6. Color

The appearance of honey is very important for consumers. The color of honey is a sensory parameter that varies between different types of honey and is dependent on chemical parameters such as mineral content and polyphenols content [48]. Regarding the mineral composition, it was argued that amber and dark honey have a higher content of certain minerals (Na, K, Ca, Mg, Fe, Cu, Zn, Al, Ni, Cd and Mn) by comparison to light-colored honey [49]. Furthermore, transition metals seem to influence the color of honey through the formation of complexes with some organic compounds. The color of honey can be also affected by both storage and thermal processing which was linked to the formation of Maillard reaction products [11].

As previously mentioned, color is a parameter that is dependent on the botanical origin of honey, as shown by the values in Table 2. The color values presented on the Pfund scale were used to classify honey by color. The color of the analyzed honey samples varied between white (rape honey), extra light amber (sunflower honey, thyme and polyfloral) and light amber (mint and raspberry honey). Manzanares et al. [50] analyzed 85 samples of honey from Tenerife, Spain and reported values between 24 and 150 mm Pfund. The color of honey samples was characterized by red and yellow shades (first quadrant of CIEL a*b* color space), as a* and b* coordinates had positive values. The lightness values (L *) of the six Tunisian honey samples analyzed by Boussaid et al. [36] ranged from 36.64 to 51.37, while in our honey samples it ranged from 34.4 to 46.1.

### 3.7. Electrical Conductivity

Another physical parameter that serves as a means to authenticate honey, and particularly the monofloral types, is electrical conductivity. Electrical conductivity is a parameter included in the new international standards regarding the differentiation between honeydew and flower honey. The limits of this parameter that were specified by standards are 500 to 800 µS·cm^−1^ for mixed honey and <500 µS·cm^−1^ in the case of pure floral honey with some exceptions [51]. Values greater than 800 µS·cm^−1^ are specific to honeydew and therefore are not acceptable for floral honey, and can confirm an adulteration with inverted sugar [52,53,54]. In the work of Kaskoniene et al. [55] it was shown that floral honey has an electrical conductivity that was lower than that of honeydew, confirming that this parameter is a quality indicator that can be used as a means to distinguish honeydew from floral honey [56].

As the values in Table 2 show, the honey samples analyzed had an electrical conductivity of less than 500 µS·cm^−1^, so they can be classified as pure floral honey. Mint honey had the highest electrical conductivity (474.05 µS·cm^−1^) followed by raspberry honey (446.16 µS·cm^−1^). Polyfloral and sunflower honey presented close values of electrical conductivity (354.09 µS·cm^−1^ and 362.27 µS·cm^−1^) and rape and thyme honey were characterized by the lowest values of electrical conductivity (162.5 µS·cm^−1^ in rape honey and 244.28 µS·cm^−1^ in thyme honey). Botanical origin had a significant influence (*p* < 0.001) on the variation of this parameter. Boussaid et al. [36] reported for Tunisian mint honey an electrical conductivity of 430 µS·cm^−1^, which was similar to our results for mint honey. In the case of thyme honey, the value reported for Tunisian honey was higher than the electrical conductivity measured for the Romanian thyme honey. Oroian and Ropciuc [44] reported, in the case of sunflower honey, an electrical conductivity value of 346.1 µS·cm^−1^ and 431.4 µS·cm^−1^ for the polyfloral honey. Usually, monofloral rape honey has low electrical conductivity, 130 to 580 µS·cm^−1^ [57] and 110–270 µS·cm^−1^ [58], which indicates that this type of honey has a lower mineral content [55]. By comparing these reported values to the values we have determined for the electrical conductivity of our rape honey samples, it can be concluded that the samples analyzed in this study were of pure rape honey.

Regarding the influence of other parameters on the electrical conductivity of honey, it was found that the variation of this parameter positively correlated with an increased ash and acid content [8]. The pollen collected by bees is a major source of minerals, and consequently, in the case of monofloral honey the electrical conductivity correlated with the pollen content [55] and may serve as a means to identify the botanical origin of honey [59]. This parameter was included in international standards to replace the ash content determination [2]. The electrical conductivity is a good criterion for identifying the botanical origin of honey and is also used for its routine control [60].

### 3.8. Total Phenolic Content

The functional properties of honey are related to the number of natural antioxidants from pollen collected by bees and other floral nectars [61]. The antioxidant effects of honey were attributed to the presence of phenolic acids, flavonoids, ascorbic acid, carotenoids, catalase, peroxidase, as well as Maillard reaction products in the composition of honey [62,63]. Table 2 presents the total phenolic content (TPC) of raspberry, mint, thyme, rape, sunflower and polyfloral honey samples. TPC varied between 18.91 mg GAE ·100 g^−1^ (thyme honey) and 23.71 mg GAE ·100 g^−1^ (mint honey); no significant differences were determined by botanical origin and year.

Chua et al. [64] reported in their study that the TPC of the analyzed honey samples ranged from 110.39 to 196.500 mg GAE ·100 g^−1^. In their study on four types of honey, Marghitaş et al. [37] reported that sunflower honey had the highest value of total polyphenol content (40 mg GAE ·100 g^−1^), while acacia honey had values between 2 and 39 mg GAE ·100 g^−1^. The total phenolic content of Indian honey was found in the range of 47 mg GAE ·100 g^−1^ of honey to 98 mg GAE/100 g of honey [51]. The TPC values determined for Romanian monofloral honey in our study were lower than those obtained by other authors when analyzing honey of different origin.

### 3.9. Flavonoids Content

The flavonoids of honey may originate from pollen, nectar or propolis [65]. In general, the main flavonoids found in honey are pinocembrin, apigenin, campferol, quercetin, pinobanksin, luteolin, galangin, hesperetin, and isorhamnetin [15]. Flavonoids have low molecular weight and are vital components of honey and its antioxidant properties [66]. Table 2 shows the values determined for flavonoids content by botanical origin and year of honey production. As in the case of total polyphenols, in this study the thyme honey samples had the lowest flavonoid content (17.45 mg QE·100 g^−1^). The highest flavonoid content was identified in raspberry honey (33.58 mg QE·100 g^−1^) followed by mint honey (25.73 mg QE·100 g^−1^), polyfloral honey (24.14 mg QE·100 g^−1^), sunflower honey (22.86 mg QE·100 g^−1^) and rape honey (20.25 mg QE·100 g^−1^). The flavonoids content was influenced by year (*p* < 0.05), but not by botanical origin (*p* > 0.05).

Mărghitaş et al. [37] reported that the total flavonoid content of honey samples ranged between 0.91–2.42 mg QE·100 g^−1^ in acacia honey, 4.70–6.98 mg QE·100 g^−1^ in tilia honey, and 11.53–15.33 mg QE·100 g^−1^ in sunflower honey. Boussaid et al. [36] reported higher total flavonoids content in mint honey (22.45 mg QE·100 g^−1^), and lower in the case of rosemary (16.24 mg QE·100 g^−1^), thyme (14.77 mg QE·100 g^−1^), orange (11.12 mg QE·100 g^−1^), horehound (11.02 mg QE·100 g^−1^), and eucalyptus (9.58 mg QE·100 g^−1^) honey.

### 3.10. DPPH Assay

The DPPH assay was used as a means to determine the free radical-scavenging activity of the honey samples. In this study the highest DPPH radical scavenging activity (Table 2) was identified for raspberry honey (79.05%) and mint honey (74.03%), and the lowest for thyme (63.77%) and rape honey (55.49%). Lachman et al. [67] also determined a lower antioxidant activity from the DPPH assay for rape honey and higher for raspberry honey in a study on honey samples from the Czech Republic. Blasa et al. [68] and Salonen et al. [69] argued that light-colored honey possessed lower antioxidant activity by comparison to darker colored honey, an observation that seems to be accurate for our study, although the differences between the free radical scavenging activities of our honey samples were not as pronounced as in the case of the above-mentioned studies.

DPPH radical scavenging activity is a parameter that varied significantly (*p* < 0.001) depending on the botanical origin of the honey samples analyzed. By comparison to the antioxidant activities reported for honey samples from other geographical regions, which include studies by Ruiz-Navajas et al. [70] who reported values of 33.4–85.5% for honey from Tabasco (Mexico) and Baltrusaityte et al. [71] who reported for honey from Lithuania values between 31.1% and 86.9%, Romanian honey had overall higher antioxidant activities.

### 3.11. Sugars Composition

Honey contains simple carbohydrates, namely glucose and fructose known as monosaccharides, which represent 65–80% of the total soluble solids, as well as 25% of other oligosaccharides (disaccharides, trisaccharides, tetrasaccharides) [72]. Determination of disaccharide content (mainly maltose and sucrose) is a tool for characterizing honey; maltose content was used to classify Spanish honey and to differentiate Brazilian honey from different geographical regions [72].

In the analyzed honey samples (Table 3), the highest fructose content was identified in thyme honey (36.77%) and the lowest in polyfloral honey (35.15%). Rape honey had the highest glucose content (31.78%) and polyfloral honey had the lowest content (24.95%) of this monosaccharide. Glucose content was the only parameter that varied significantly (*p* < 0.01) depending on the botanical origin of the analyzed samples. Maltose (maximum value of 1.79% in polyfloral honey), trehalose (maximum value of 2.35% in rape honey) and melesitose (maximum value of 1.34% in thyme honey) were sugars that together with fructose and glucose were found in significant concentrations in the analyzed honey samples. Sucrose and rafinose had values between 0.07% (raspberry honey) and 0.73% (polyfloral honey), respectively 0.21% (rape honey) and 0.42% (polyfloral honey). Apart from individual sugars, for all 45 honey samples, the fructose/glucose ratio was also calculated. When the content of fructose is higher than that of glucose honey is fluid, thus, this ratio can be used to identify the crystallization state of honey [73,74]. Suarez et al. [75] reported that the fructose/glucose ratio might also impact the flavor of honey since fructose is sweeter than glucose. All honey samples examined were fluid, as the fructose/glucose ratio was greater than 1 (Table 2).

Some authors argued that the amount and ratio of specific carbohydrates, such as fructose, glucose and oligosaccharides can be used to identify whether honey is monofloral or polyfloral [76]. Kaskonien & Venskutonis [55] considered that the use of carbohydrates as floral markers is not often preferred because of the difficulties encountered in identifying one or more sugars contained by honey. Cotte et al. [76] analyzed authentic monofloral honey samples and found differences in the carbohydrate composition based on botanical origin. Fir honey samples were high in trisaccharides, in particular raffinose (2.1%), melesitose (5.7%) and erlose (2.1%). In contrast, in rape and sunflower honey these trisaccharides were absent, which serves as a way to distinguish them from other botanical varieties. Acacia honey has a high concentration of trisaccharides (1.9%), with erlose being the predominant trisaccharide in this type of honey; lavender and tilia honey were characterized by lower concentrations of erlose (1.4 and 1.0% respectively) [76].

### 3.12. Polyphenols Composition

Polyphenols are powerful antioxidants that can reach more than 0.8% (by weight) in bee products [77]. Phenolic acids and flavonoids were extensively investigated in honey [78] and were used to evaluate its quality. The correlations between antioxidant activity and total concentration of phenols was confirmed for seven types of honey from Italy [79] and four honey types from Romania [37]. In another study on Portuguese honey, it was shown that polyphenols in honey were responsible for its antimicrobial effects [80]. Phenolic compounds can be used to classify honey according to its botanical origin [78]. The composition of honey in polyphenols was found to be mostly dependent on the botanical origin due to the fact that these compounds mostly originate from the nectar collected by bees; the nature and quantity of phenolic compounds can also vary with the season, climatic conditions and processing factors [63,79].

In the studied samples, 12 polyphenols were analyzed, which were mostly found in all samples in different concentrations (Table 4). Gallic acid was found at a high concentration (1.55 mg·100 g^−1^) in mint honey, while the lowest value was identified in thyme honey (0.57 mg·100 g^−1^). The protocatechuic and 4-hydroxybenzoic acids were identified in higher concentrations in mint (2.04 mg protocatechuic acid·100 g^−1^ and 1.20 mg 4-hydroxybenzoic acid·100 g^−1^) and raspberry honey (2.57 mg protocatechuic acid·100 g^−1^ and 2.33 mg 4-hydroxybenzoic acid·100 g^−1^). Compared to other honey types, mint also had a high content of vanillic acid (3.03 mg·100 g^−1^) and chlorogenic acid (1.48 mg·100 g^−1^). These two phenolic acids were also found in sunflower and thyme honey in large quantities. Caffeic acid predominated in polyfloral honey (1.20 mg·100 g^−1^) and did not exceed the level of 0.38 mg·100 g^−1^ in other honey samples. Thyme honey had the highest content of *p*-coumaric acid, while myricetin predominated in rape honey, although it was found in all types of honey. Rosmarinic acid was found only in raspberry honey at a very small concentration of 0.03 mg·100 g^−1^, while kaempferol was determined only in polyfloral honey in a concentration of 0.38 mg·100 g^−1^. Quercetin was quantified only in 3 types of honey: mint, polifloral and sunflower honey and luteolin was not determined in any sample.

Gasic et al. [81] observed that quercetin and eriodictiol can be used for sunflower honey authentication and we observed too that quercetin is presented in the sunflower honey analyzed.

### 3.13. Organic Acids Composition

Organic acids are found in honey in small quantities (<0.5%), but are important chemical components because of their significant contribution to the stability and preservation of the physicochemical and sensory properties of honey [11].

The total acid content increases due to the fermentation phenomena and aging that may occur during storage [82]. Some authors have suggested that organic acid profiles are useful for identifying the botanical and/or geographical origin of honey [83].

As shown in Table 5, the predominant acid in all the honey samples analyzed was gluconic acid. The maximum gluconic acid content was determined in raspberry honey (4.83 g·kg^−1^) and the lowest value in rape honey (3.59 g·kg^−1^). Brugnerotto et al. [84] also identified gluconic acid as the predominant acid in all the honey samples that they studied. Gluconic acid is predominant in both honeydew and floral honey and its concentration can be influenced by the botanical source and the pollen and nectar of the flowers collected by bees. In our study, the concentration of gluconic acid was not influenced by botanical origin or year of production (*p* > 0.05).

Romanian mint and thyme honey were also high in propionic acid (2.67 g·kg^−1^ and 2.36 g·kg^−1^). Honey samples had a succinic acid content that ranged from a minimum value of 0.05 g·kg^−1^ in raspberry honey to a maximum value of 0.13 g·kg^−1^ in mint honey. In the study conducted by Suarez-Luque et al. [85] on 50 honey samples from Galicia (Santiago de Compostela, Spain), the succinic acid content was much higher. Formic, acetic, lactic and butyric acid were determined in low concentrations in all honey samples. The content of honey samples in both propionic and acetic acids was strongly influenced (*p* < 0.001) by botanical origin.

The quantification of malonic and glycolic acids in floral honey was firstly reported in a study by Brugnerotto et al. [84] who determined concentrations of 82.2–134 mg malonic acid·100 g^−1^ and 27.8–43.7 mg glycolic acid·100 g^−1^. Acetic, lactic, formic, and propionic acids were identified in lower concentrations, while fumaric and tartaric acids were not detected. In their study, citric and malic acid concentrations were of 48.2–506 mg·100 g^−1^ and 19.9–132 mg·100 g^−1^, respectively [84].

Suarez-Luque et al. [85] also observed variations in the composition of organic acids in honey that were attributed to its botanical origin. The concentration of citric, malic, succinic and fumaric acid was high in chestnut honey and low in eucalyptus honey. Polyfloral honey had a high content of maleic acid, while clover honey did not contain malic and succinic acids.

The concentration and content of organic acids, as well as ketones and benzene compounds such as 2-hydroxy-2-propanone, 2-phenylethanol, butanoic acid or benzyl alcohol, which were identified in fresh honey, increase with temperature and storage time [86].

### 3.14. Principal Component Analysis (PCA)

**Figure 1:**
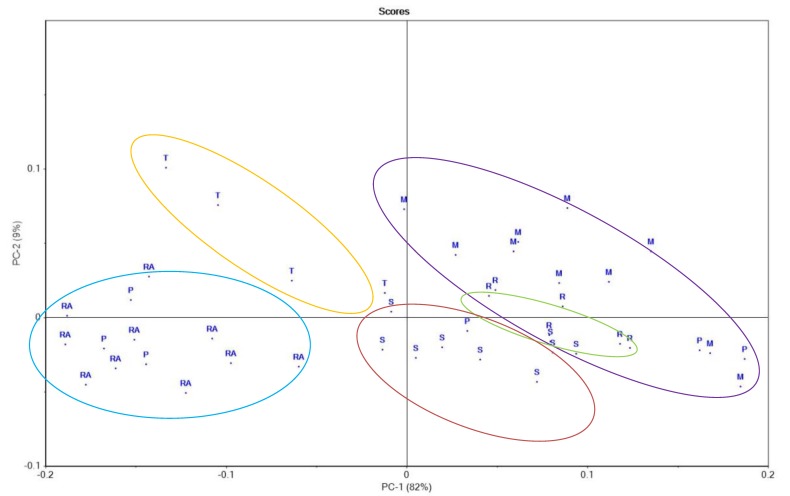
Principal component analysis—scores: RA—rape, T—thyme, P—polyfloral, S—sunflower, M—mint, and R—raspberry honey. Blue ellipse—rape honey group, yellow ellipse—thyme honey group, red ellipse—sunflower honey group, green ellipse—raspberry honey group, and purple ellipse—mint honey group.

Principal component analysis (PCA) is a statistical procedure that is used to perform a comparison of the results of analytical methods applied to a group of samples. In this study, PCA was applied to analyze and identify the honey samples that share similar characteristics from a total number of 45 samples of different honey types from various regions in Romania. The first principal component (PC-1) accounted for 82% of the variance, while the second principal component (PC-2) accounted for 9% of the variance; together, the first two principal components accounted for 91% of the initial variability. The separation of the honey samples according to botanical origin is shown in Figure 1. As seen in Figure 1, there are three ellipses which represent the rape, sunflower and thyme honey, which are not overlapped with other honey samples, except the polyfloral honey. Regarding the mint and raspberry honey, it can be observed that the raspberry honey ellipse is placed in the mint honey ellipse so a clear separation cannot be observed in this sample. Polyfloral honey was not perfectly grouped due to the fact that this honey type has a wide variety of pollen grains.

**Figure 2:**
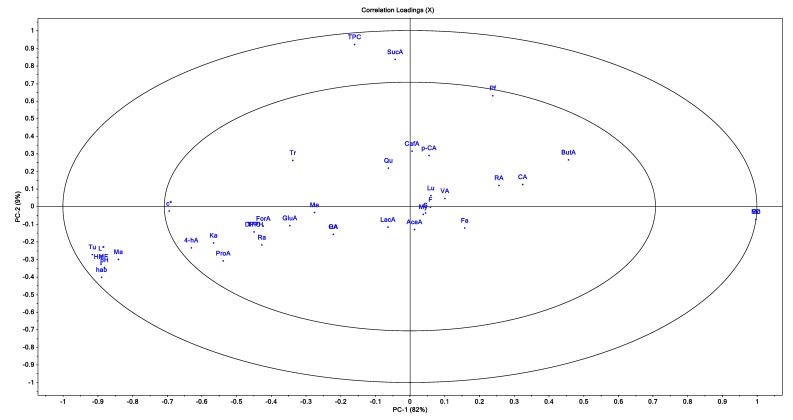
Principal component analysis—loadings: L*, h*_ab_, c*_ab_, Pf—Pfund color, pH, Fa—free acidity, EC—electrical conductivity, Mo—moisture, HMF, TPC—total polyphenols content, TFC—total flavonoids content, DPPH, GA—gallic acid, PA—protocatechuic acid, 4-hA—4-Hydroxybenzoic acid, VA—vanillic acid, CA—chlorogenic acid, CafA—caffeic acid, p-CA—p-coumaric acid, RA—rosmarinic acid, My—miricetin, Qu—quercetin, Lu—luteolin, Ka—kaempferol, F—fructose content, G—glucose, S—sucrose, Tu—turanose, Ma—manose, Tr—trehalose, Me—melesitose, Ra—raffinose, GluA—gluconic acid, ForA—formic acid, AcetA—acetic acid, ProA—propionic acid, LacA—lactic acid, ButA—butyric acid, and SucA—succinic acid.

In Figure 1, the honey types are marked as: RA—rape, T—thyme, P—polyfloral, S—sunflower, M—mint, and R—raspberry honey. In Figure 2, the parameters used for the projection are abbreviated as: Pf—Pfund color, pH, Fa—free acidity, EC—electrical conductivity, Mo—moisture, HMF, TPC—total polyphenols content, TFC—total flavonoids content, DPPH, GA—gallic acid, PA—protocatechuic acid, 4-hA—4-Hydroxybenzoic acid, VA—vanillic acid, CA—chlorogenic acid, CafA—caffeic acid, p-CA—p-coumaric acid, RA—rosmarinic acid, My—miricetin, Qu—quercetin, Lu—luteolin, Ka—kaempferol, F—fructose content, G—glucose, S—sucrose, Tu—turanose, Ma—manose, Tr—trehalose, Me—melesitose, Ra—raffinose, GluA—gluconic acid, ForA—formic acid, AcetA—acetic acid, ProA—propionic acid, LacA—lactic acid, ButA—butyric acid, and SucA—succinic acid.

In Figure 2, the parameters which are in the outer ellipse have a greater contribution to variability than the parameters located in the inner ellipse. The rape honey samples were correlated with L* values, pH, c*_ab_, h*_ab_, turanose content, manose content and HMF content. The thyme honey samples were correlated with trehalose content and mint honey with caffeic acid, *p*-coumaric acid, vanillic acid, rosmarinic acid and chlorogenic acid content. Regarding the physicochemical parameters, it seems that the moisture content was in opposition to the rest of the parameters.

There was a clear differentiation between Mo variable, the variable groups Tu, L*, Ma, HMF, pH and h*_ab_ (PC-1 direction) and variable groups TPC and SucA (PC-2 direction). Between variable groups from the PC-1 direction (Tu, L*, Ma, HMF, pH, h*_ab_ and M), there was no correlation with variables TPC and SucA. Between Tu, L*, HMF, pH and h*_ab,_ and variable Mo, there was negative correlation and the highest fraction of explained variance among these variables was 82%. Furthermore, the small distance between Tu, L*, HMF, Ma and h*_ab_ showed a strong correlation between variables.

## 4. Conclusions

The physicochemical parameters of raspberry, mint, sunflower, thyme, rape and polyfloral honey samples from different regions in Romania were analyzed in order to examine their usefulness in the classification of honey according to botanical origin. All honey samples had pH and free acidity values in the limits permitted by quality standards, which confirm the freshness of all honey samples. With the exception of three samples (two samples of mint honey and one sample of thyme honey) that had HMF content above the allowed limit, all the samples that were analyzed in this study met the quality requirements for honey. For most of the physicochemical parameters (color parameters, electrical conductivity, HMF content, DPPH, free acidity and pH) the differences between the measured levels were determined by the botanical origin of honey. The sugar composition, individual phenolic compounds and organic acids composition of honey varied to some extent between samples, however, these parameters were not influenced by the botanical origin of honey. As a consequence, no compound that can be used as a chemical marker was identified. PCA analysis was successful in the rape, sunflower and thyme honey samples while the mint and raspberry honey have not been clearly separated, but for a better classification of unknown honey it is necessary to increase the number of samples analyzed.

## Figures and Tables

**Table 1:** Pollen types in honey samples.

Honey Type	Principal Pollen Type (min.%–max.%)
Raspberry	*Rubus idaeus* (49.1–82.3%)
Rape	*Brassica* spp. (50.1–71.1%)
Sunflower	*Helianthus* spp. (46.5–92.1%)
Mint	*Mentha* spp. (46.5–65.1%)
Thyme	*Thymus* spp. (22–45%)

**Table 2:** Physicochemical properties for different types of honey (raspberry, mint, rape, thyme, polyfloral and sunflower). Mean values and standard deviation in brackets.

Parameter	Origin						F Value	Year		F Value
	Mint	Polyfloral	Rape	Raspberry	Sunflower	Thyme		2017	2018	
L *	35.3 (6.24) ^bc^	46.1 (3.67) ^a^	41.4 (3.48) ^a^	34.4 (3.36) ^c^	41.05 (6.93) ^a^	43.2 (6.31) ^ab^	5.05 ***	40.5 (5.58) ^a^	39.3 (7.03) ^a^	1.67 ^ns^
h_ab_	65.6 (9.38) ^d^	84.7 (10) ^b^	96.6 (6.03) ^a^	73.4 (8.43) ^c^	83.4 (3.17) ^b^	81.8 (4.76) ^bc^	20.30 ***	79.6 (12.56) ^a^	82.2 (13.48) ^a^	1.56 ^ns^
c_ab_	19.7 (3.42) ^c^	26.2 (5.37) ^ab^	19.2 (6.66) ^c^	23.8 (1.78) ^bc^	27.2 (5.81) ^a^	29.8 (4.48) ^a^	7.71 ***	26.3 (5.43) ^a^	21.1 (5.72) ^b^	24.85 ***
Pfund (mm Pfund)	74.3 (14.54) ^a^	40.9 (20.41) ^cd^	29.4 (11.16) ^d^	61.4 (14.35) ^ab^	37.6 (10.23) ^cd^	50.1 (12.29) ^bc^	13.28 ***	49.4 (21.19) ^a^	48.1 (21.96) ^a^	0.01 ^ns^
pH	4.20 (0.25) ^a^	4.09 (0.24) ^ab^	4.22 (0.08) ^a^	4.16 (0.12) ^a^	3.94 (0.25) ^b^	3.91 (0.19) ^ab^	2.51 *	4.08 (0.25) ^a^	4.1 (0.21) ^a^	0.67 ^ns^
Free acidity (meq·kg^−1^)	26.9 (8.89) ^a^	23.9 (12.54) ^ab^	16 (4.43) ^b^	27.3 (7.71) ^a^	31.6 (12.20) ^a^	22.5 (8.16) ^ab^	3.07 **	22.01 (8.69) ^a^	26.8 (11.12) ^a^	2.28 ^ns^
EC (μS·cm^−1^)	474 (92.76) ^a^	354 (242.77) ^abc^	162 (38.26) ^d^	446 (68.57) ^ab^	362 (55.03) ^bc^	244(54.13) ^cd^	10.71 ***	310 (154.86) ^a^	367 (151.89) ^a^	2.28 ^ns^
Moisture (%)	17.7 (1.10) ^b^	19.6 (1.65) ^a^	18.4 (0.86) ^ab^	18.3 (1.05) ^ab^	18.4 (1.48) ^ab^	17.3 (1.95) ^b^	1.71 ^ns^	17.9 (1.01) ^a^	18.6 (1.56) ^a^	3.76 *
HMF (mg·kg^−1^)	29.2 (23.22) ^a^	10 (8.84) ^b^	13.3 (14.10) ^b^	18.7 (16.33) ^b^	8.26 (4.49) ^b^	30.8 (20.96) ^a^	6.24 ***	28.4 (26.31) ^a^	20.2 (26.26) ^a^	0.72 ^ns^
TPC (mg GAE·100 g^−1^)	23.7 (4.37) ^a^	20.3 (7.67) ^a^	19.9 (4.83) ^a^	19.9 (4.83) ^a^	21.1 (7.18) ^a^	18.9 (3.82) ^a^	0.35 ^ns^	21.4 (5.83) ^a^	20.5 (5.98)^a^	1.15 ^ns^
FC (mg QE·100 g^−1^)	25.7 (10.55) ^b^	24.1 (5.76) ^b^	20.2 (12.21) ^b^	33.5 (6.62) ^a^	22.8 (8.73) ^b^	17.4 (9.33) ^b^	2.29 ^ns^	21.1 (10.42) ^a^	26.3 (9.46)^a^	4.26 *
DPPH (%)	74.03 (5.84) ^ab^	70.7 (15.90) ^ab^	55.4 (6.88) ^c^	79.05 (13.51) ^a^	68.03 (8.01) ^b^	67.3 (9.82) ^ab^	5.24 ***	67.3 (13.12) ^a^	69.1 (11.38)^a^	0.68 ^ns^

^ns^ not significant (*p* > 0.05), * *p* < 0.05, ** *p* < 0.01, *** *p* < 0.001, ^a–d^ different letters in the same row indicate significant differences between samples (*p* < 0.001) according to the LSD test with *α* = 0.05. Pfund—color in Pfund scale, EC—electrical conductivity, HMF—5-hydroxymethylfurfural, TPC—total phenolic content, FC—flavonoids content, DPPH—radical scavenging activity.

**Table 3:** Sugars content for different types of Romanian honey. Mean values and standard deviation in brackets.

Sugars (%)	Origin						F Value	Year		F Value
	Mint	Polyfloral	Rape	Raspberry	Sunflower	Thyme		2017	2018	
Fructose	36.03 (2.33)^a^	35.15 (1.45) ^a^	35.26 (1.28) ^a^	36.30 (1.43) ^a^	36.74 (1.73) ^a^	36.77 (3.79) ^a^	0.8 ^ns^	36.66 (2.27) ^a^	35.45 (1.51) ^a^	4.08 ^ns^
Glucose	27.87 (2.81) ^bc^	24.95 (1.27) ^c^	31.78 (2.71) ^a^	29.00 (2.72) ^ab^	28.37 (3.97) ^b^	26.86 (2.80) ^bc^	4.66 **	28.4 (3.96) ^a^	28.6 (3.11) ^a^	0.57 ^ns^
Sucrose	0.45 (1.09) ^a^	0.73 (1.23) ^a^	0.08 (0.20) ^a^	0.07 (0.07) ^a^	0.35 (0.53) ^a^	0.49 (0.78) ^a^	0.68 ^ns^	0.33 (0.74) ^a^	0.34(0.77) ^a^	0.13 ^ns^
Turanose	0.42 (0.19) ^a^	0.2 (0.11) ^a^	0.66 (1.24) ^a^	0.29 (0.10) ^a^	0.38 (0.30) ^a^	0.31 (0.26) ^a^	0.86 ^ns^	0.53 (0.88) ^a^	0.31 (0.20) ^a^	1.17 ^ns^
Maltose	1.44 (0.49) ^a^	1.79 (0.40) ^a^	1.82 (1.52) ^a^	1.32 (0.40) ^a^	1.62 (0.93) ^a^	1.48 (0.84) ^a^	0.42 ^ns^	1.76 (1.12) ^a^	1.46 (0.66) ^a^	1.86 ^ns^
Trehalose	1.45 (0.83) ^a^	1.87 (0.58) ^a^	2.35 (3.22) ^a^	1.57 (0.58) ^a^	1.92 (1.03) ^a^	2.07 (0.73) ^a^	0.33 ^ns^	2.1 (2.29) ^a^	1.68 (0.83) ^a^	0.68 ^ns^
Melesitose	1.03 (0.31) ^a^	1.10 (0.23) ^a^	1.08 (0.69) ^a^	0.96 (0.28) ^a^	1.06 (0.48) ^a^	1.34 (0.84) ^a^	0.36 ^ns^	1.21 (0.59) ^a^	0.97 (0.35) ^a^	3.47 ^ns^
Raffinose	0.31 (0.15) ^ab^	0.42 (0.12) ^a^	0.21 (0.11) ^b^	0.36 (0.21) ^ab^	0.40 (0.27) ^ab^	0.40 (0.28) ^ab^	1.63 ^ns^	0.34 (0.21) ^a^	0.33 (0.19) ^a^	0.36 ^ns^
F/G ratio	1.30 (0.14) ^a^	1.40 (0.03) ^a^	1.11 (0.11) ^b^	1.26 (0.14) ^ab^	1.33 (0.29) ^a^	1.38 (0.22) ^a^	3.06 *	1.32 (0.24) ^a^	1.25 (0.14) ^a^	2.68 ^ns^

^ns^ not significant (*p* > 0.05), * *p* < 0.05, ** *p* < 0.01, ^a–c^ different letters in the same row indicate significant differences between samples (*p* < 0.01), according to LSD test with *α* = 0.05. F—fructose, G—glucose.

**Table 4:** Polyphenols content for different types of Romanian honey. Mean values and standard deviation in brackets.

Polyphenols (mg·100 g^−1^)	Origin						F Value	Year		F Value
	Mint	Polyfloral	Rape	Raspberry	Sunflower	Thyme		2017	2018	
Gallic acid	1.55 (1.71) ^a^	1.03 (0.86) ^a^	0.65 (0.27) ^a^	0.95 (0.45) ^a^	0.83 (0.46) ^a^	0.57 (0.24) ^a^	1.24 ^ns^	1.00 (1.31) ^a^	0.94 (0.50) ^a^	0.14 ^ns^
Protocatechuic acid	2.04 (2.52) ^ab^	0.71 (1.20) ^bc^	0.44 (0.50) ^c^	2.57 (1.47) ^a^	1.37 (0.41) ^abc^	1.77 (1.05) ^abc^	2.61 *	1.21 (1.26) ^a^	1.58 (1.77) ^a^	0.69 ^ns^
4-Hydroxybenzoic acid	1.20 (1.03) ^ab^	0.52 (0.53) ^b^	0.41 (0.21) ^b^	2.33 (3.09) ^a^	1.15 (0.9) ^ab^	0.83 (0.77) ^ab^	1.70 ^ns^	0.87 (0.82) ^a^	1.18 (1.70) ^a^	0.96 ^ns^
Vanillic acid	3.03 (3.05) ^a^	1.24 (2.49) ^ab^	0.17 (0.46) ^b^	1.83 (3.10) ^ab^	2.35 (2.69) ^ab^	1.62 (2.72) ^ab^	1.05 ^ns^	1.56 (2.99) ^a^	1.87 (2.24) ^a^	0.04 ^ns^
Chlorogenic acid	1.48 (2.56) ^a^	1.16 (1.94) ^a^	0.004 (0.01) ^a^	0.45 (0.73) ^a^	0.36 (0.67) ^a^	1.41 (2.83) ^a^	0.93 ^ns^	0.31 (1.26) ^a^	1.08 (1.90) ^a^	1.28 ^ns^
Caffeic acid	0.23 (0.24) ^a^	1.20 (2.72) ^a^	0.18 (0.06) ^a^	0.38 (0.49) ^a^	0.30 (0.32) ^a^	0.22 (0.30) ^a^	0.70 ^ns^	0.22 (0.25) ^a^	0.51 (1.33) ^a^	0.85 ^ns^
*P*-coumaric acid	0.61 (0.53) ^a^	0.70 (0.68) ^a^	0.46 (0.32) ^a^	0.74 (0.85) ^a^	0.80 (0.41) ^a^	1.06 (1.07) ^a^	0.49 ^ns^	0.60 (0.57) ^a^	0.75 (0.61) ^a^	0.55 ^ns^
Rosmarinic acid	0 ^a^	0 ^a^	0 ^a^	0.03 (0.09) ^a^	0 ^a^	0 ^a^	1.07 ^ns^	0.01 (0.05) ^a^	0 ^a^	0.44 ^ns^
Miricetin	1.86 (0.87) ^a^	1.73 (1.40) ^a^	2.23 (1.03) ^a^	1.02 (1.16) ^a^	1.37 (1.21) ^a^	1.99 (1.58) ^a^	1.01 ^ns^	1.50 (1.23) ^a^	1.90 (1.09) ^a^	0.4 ^ns^
Quercetin	0.30 (0.41) ^a^	0.99 (2.28) ^a^	0 ^a^	0 ^a^	0.19 (0.30) ^a^	0 ^a^	1 ^ns^	0 ^a^	0.43 (1.13) ^a^	1.85 ^ns^
Luteolin	0	0	0	0	0	0	−	0	0	−
Kaempferol	0	0.38 (0.94) ^a^	0	0	0	0	1.09 ^ns^	0	0.09 (0.46) ^a^	0.51 ^ns^

^ns^ not significant (*p* > 0.05), * *p* < 0.05; ^a–c^ different letters in the same row indicate significant differences between samples (*p* < 0.05), according to the LSD test with *α* = 0.05.

**Table 5:** Organic acids content for different types of Romanian honey. Mean values and standard deviation in brackets.

Organic Acids (g·kg^−1^)	Origin						F Value	Year		F Value
	Mint	Polyfloral	Rape	Raspberry	Sunflower	Thyme		2017	2018	
Gluconic acid	4.46 (1.53) ^ab^	4.21 (0.48) ^ab^	3.59 (1.12) ^b^	4.83 (0.34) ^a^	4.76 (0.55) ^a^	4.50 (0.49) ^ab^	1.60 ^ns^	4.11 (1.40) ^a^	4.53 (0.53) ^a^	1.57 ^ns^
Formic acid	0.37 (0.43) ^ab^	0.18 (0.09) ^b^	0.21 (0.17) ^b^	0.28 (0.27) ^ab^	0.53 (0.36) ^ab^	0.77 (1.01) ^a^	1.60 ^ns^	0.30 (0.31) ^a^	0.42 (0.49) ^a^	1.15 ^ns^
Acetic acid	0.77 (0.30) ^a^	0.39 (0.22) ^bc^	0.18 (0.05) ^c^	0.58 (0.20) ^ab^	0.4 (0.26) ^bc^	0.3 (0.08) ^bc^	6.99 ***	0.41 (0.25) ^a^	0.47 (0.33) ^a^	0.75 ^ns^
Propionic acid	2.67 (1.52) ^a^	0.72 (0.26) ^b^	0.62 (0.42) ^b^	0.86 (0.49) ^b^	0.79 (0.28) ^b^	2.36 (0.29) ^a^	11.36 ***	1.48 (1.09) ^a^	1.17 (1.20) ^a^	1.11 ^ns^
Lactic acid	0.18 (0.28) ^b^	0.12 (0.22) ^b^	0.14 (0.26) ^b^	0.09 (0.07) ^b^	0.14 (0.20) ^b^	0.59 (0.52) ^a^	1.66 ^ns^	0.18 (0.26) ^a^	0.17 (0.30) ^a^	0 ^ns^
Butyric acid	0.51 (0.42) ^a^	0.87 (1.61) ^a^	0.07 (0.11) ^a^	0.11 (0.16) ^a^	0.32 (0.38) ^a^	0.23 (0.20) ^a^	1.25 ^ns^	0.28 (0.33) ^a^	0.39 (0.84) ^a^	0.18 ^ns^
Succinic acid	0.13 (0.12) ^a^	0.1 (0.19) ^a^	0.09 (0.14) ^a^	0.05 (0.11) ^a^	0.11 (0.09) ^a^	0.08 (0.07) ^a^	0.24 ^ns^	0.09 (0.13) ^a^	0.10 (0.12) ^a^	0.01 ^ns^

^ns^ not significant (*p* > 0.05), *** *p* < 0.001; ^a–c^ different letters in the same row indicate significant differences between samples (*p* < 0.05), according to the LSD test with *α* = 0.05.
